# Electroencephalographic Cross-Frequency Coupling as a Sign of Disease Progression in Patients With Mild Cognitive Impairment: A Pilot Study

**DOI:** 10.3389/fnins.2020.00790

**Published:** 2020-08-11

**Authors:** Christian Sandøe Musaeus, Malene Schjønning Nielsen, Jørgen Sandøe Musaeus, Peter Høgh

**Affiliations:** ^1^Department of Neurology, Danish Dementia Research Centre (DDRC), Rigshospitalet, University of Copenhagen, Copenhagen, Denmark; ^2^Department of Neurology, Regional Dementia Research Centre, Zealand University Hospital, Roskilde, Denmark; ^3^Niels Bohr Institute, University of Copenhagen, Copenhagen, Denmark; ^4^Department of Clinical Medicine, University of Copenhagen, Copenhagen, Denmark

**Keywords:** mild cognitive impairment, Alzheimer’s disease, cross-frequency coupling, phase-amplitude coupling, gamma, theta

## Abstract

Mild cognitive impairment (MCI) refers to mild objective cognitive deficits and is associated with the later development of Alzheimer’s disease (AD). However, not all patients with MCI convert to AD. EEG spectral power has shown promise as a marker of progression, but brain oscillations in different frequencies are not isolated entities. Coupling between different frequency bands, so-called cross-frequency coupling (CFC), has been associated with memory function and may further contribute to our understanding of what characterizes patients with MCI who progress to AD. In the current study, we wanted to investigate the changes in gamma/theta CFC in patients with AD and MCI compared to HC and in patients with pMCI compared to patients with sMCI. Furthermore, we wanted to investigate the association with cognitive test scores. EEGs were included at baseline for 15 patients with AD, 25 patients with MCI, and 36 older HC, and the participants were followed for up to 3 years. To investigate CFC, we calculated the modulation index (MI), which has been shown to be less affected by noisy data compared to other techniques. We found that patients with pMCI showed a significantly lower global gamma/theta CFC compared to patients with sMCI. In addition, global gamma/theta CFC was significantly correlated with Addenbrooke’s Cognitive Examination (ACE) score (*p*-value = 0.030, rho = 0.527). Although not significant, patients with AD and MCI showed a lower gamma/theta CFC compared to HC. These findings suggest that gamma/theta CFC is important for proper cognitive functioning and that a decrease in gamma/theta CFC in patients with MCI may be a sign of progression. Gamma/theta CFC may therefore serve as a progression marker in MCI, but larger studies are needed to validate these findings.

## Introduction

Alzheimer’s disease (AD) is a progressive neurodegenerative disease leading to a disruption of normal brain oscillations (Coben et al., 1985; Jeong, 2004; Musaeus et al., 2018a). The disruption of brain oscillations has even been seen in the prodromal phase of a disease known as mild cognitive impairment (MCI) (Musaeus et al., 2018b). MCI refers to mild objective cognitive deficits and is associated with the later development of AD (Petersen et al., 1999; Petersen, 2004), but not all patients with mild MCI convert to AD (Petersen, 2004). Attempts have been made to use electroencephalography (EEG) as a marker of whether a patient with MCI will progress (pMCI) or remain stable (sMCI) using spectral power (Jelic et al., 2000; Poil et al., 2013; Musaeus et al., 2018b). However, brain oscillations of different frequencies are not isolated entities, and the coupling between different frequency bands may further our understanding of the pathophysiological processes (Buzsáki and Watson, 2012). This coupling between different frequencies, a phenomenon called cross-frequency coupling (CFC) (Canolty and Knight, 2010), has shown a variety of functional roles, including memory in both rodents and humans.

The most commonly studied coupling is between the amplitude in the gamma band and the phase in the theta range (gamma/theta). This has been associated with activity in the hippocampus and performance in memory tasks in rodents (Tort et al., 2009; Canolty and Knight, 2010; Lisman and Jensen, 2013) and has been shown to be vital for working memory in humans (Axmacher et al., 2010). In rodent models of AD, studies have found that impaired gamma/theta coupling arises before amyloid beta accumulation (Goutagny et al., 2013) and attenuated gamma/theta coupling in knock-out mice (Zhang et al., 2016; Bazzigaluppi et al., 2018). Studies investigating patients with AD have found diverging results (Poza et al., 2017; Wang et al., 2017). One study found a decrease in CFC (Poza et al., 2017), while another found an increased CFC (Wang et al., 2017) in AD compared to healthy controls (HC). Furthermore, one study found that CFC was decreased in both AD and MCI compared with HC and associated with working memory deficits (Goodman et al., 2018). The underlying reason for the diverging results may be due to differences in the methods applied to calculate CFC. Furthermore, to our knowledge, CFC has not been examined in patients with pMCI compared to patients with sMCI, which may give valuable knowledge on the underlying pathophysiology.

When considering the CFC in different frequency bands, besides gamma/theta CFC, studies have found that CFC coupling between alpha, beta, and gamma may play an important role in coordination in perception, consciousness, and working memory (Palva and Palva, 2007). Furthermore, beta–delta CFC has been associated with neuropsychiatric symptoms, which is also prevalent in patients with AD (Miskovic et al., 2010). Therefore, couplings between other frequencies besides gamma/theta CFC may also be disrupted in both AD and MCI.

In the current study, we investigated the differences in gamma/theta CFC between AD, MCI, and HC and the differences in patients with pMCI compared to patients with sMCI. Our hypothesis was that patients with AD showed lower gamma/theta CFC compared to both MCI and HC and that pMCI showed lower gamma/theta CFC than sMCI. In an exploratory manner, we also examined the changes in CFC in multiple frequency bands. Lastly, we investigated the association between gamma/theta CFC and both cognitive function and cerebrospinal fluid (CSF) markers in AD.

## Materials and Methods

### Subjects

Data have also been presented in other studies using other types of analyses (Musaeus et al., 2018b, 2019a,b; Schjonning Nielsen et al., 2018), and the data were in part collected as a large multicenter study (Engedal et al., 2015). This cohort study was conducted at two Danish memory clinics and involved patients who were diagnosed with either MCI or mild AD and a baseline Mini-Mental State Examination (MMSE) score of ≥22. The following exclusion criteria were used: (1) no close relatives who wished to participate, (2) participating in intervention studies or (3) suffered from other neurological, psychiatric, or other severe diseases, (4) received sedative medication, and (5) had any past or current addiction to alcohol or medications.

The HC were volunteers and recruited through public advertisements at memory clinics, at local associations for elders, or through an online recruitment site. The inclusion criteria were (1) between 50 and 90 years old, (2) MMSE score >26, (3) ACE > 85, (4) normal neurological and clinical examination, (5) normal or age-related brain atrophy measured on a computed tomography scan, and (6) normal routine blood tests. The exclusion criteria were (1) an inability to participate (including impaired vision or hearing), (2) cognitive symptoms including memory complaints, (3) major neurological, psychiatric, or other severe diseases, which could cause cognitive impairments including major depression or a geriatric depression scale score >7, (4) pregnancy, (5) had undergone general anesthesia, (6) received electroconvulsive therapy in the last 3 months, (7) use of sedatives, or (8) past or current addiction to alcohol or medications.

In total, we included 17 patients with AD, 27 patients with MCI, and 38 HC in the study. The study was approved by the Regional Ethical Committee.

Due to excessive artifacts, we excluded the following number of EEGs: two from patients with AD, two from patients with MCI, and one from HC. When comparing pMCI and sMCI, one EEG from MCI was excluded due to clinical progression to vascular dementia. One HC was excluded from the CFC analysis due to fewer than 70 1-s epochs.

### Diagnostic Assessment

All the patients underwent a full physical and neurological examination, routine blood analysis, brain CT or MRI scan as well as cognitive screening. The cognitive screening included MMSE, Addenbrooke’s Cognitive Examination (ACE), and Digit Symbol Substitution Test (DSST), including Clinical Dementia Rating (CDR). In addition, the patients and the relatives underwent NeuroPsychiatric Inventory (NPI), Major Depression Inventory (MDI), and Activities of Daily Living Inventory (ADCS-ADL). All CT and MRI scans were examined by a neuro-radiologist. Except for two patients with MCI and six HC, all patients had a lumbar puncture performed. If it was considered relevant for the diagnostic evaluation, the patients underwent a neuropsychological evaluation by a clinical neuropsychologist. Diagnoses were settled by consensus of a multidisciplinary team based on all examination results. The included MCI patients fulfilled the Winblad consensus criteria (Winblad et al., 2004) and the AD patients fulfilled the NIA-AA criteria (McKhann et al., 2011).

All HC underwent the standardized diagnostic assessment at the time of inclusion, which included cognitive tests (ACE, MMSE, and DSST) and MDI. Lumbar puncture and subsequent CSF analysis was performed on almost all HC. At the baseline visit, all HC were referred for a standardized EEG, and the EEG recordings were not used in the assessment.

### Study Design

All patients were recruited within 6 months after the diagnosis and tests were repeated at inclusion. Follow-up visits were carried out on a yearly basis as part of the routine visits, with serial cognitive tests, which included MMSE and ACE and the NPI, MDI, ADCS-ADL, and CDR scales. Clinical progression of MCI to AD was determined based on whether the patient clinically fulfilled the NIA-AA criteria (McKhann et al., 2011). If a patient with MCI progressed to another diagnosis than AD, they were excluded from the comparison between pMCI and sMCI.

The primary investigator performing the tests was blinded from the results of the EEG, imaging, and CSF analysis during the study period. This was done to ensure that the investigator was blinded for the potential presence of underlying AD pathology.

The reason for the dropout from the study without having a yearly follow-up for 3 years was mostly that they were recruited later and therefore could not complete all 3 years (six AD and eight MCI) or were not able to be tested in a proper way in a follow-up session (three AD and one MCI), wanted to drop out (one AD, two MCI, and three HC) or died (one AD and two MCI).

### Electroencephalography Recording

The EEG recordings were performed at the two participating centers and performed using NicoletOne EEG Systems (Natus^®^), with a sampling rate of either 500 or 1,000 Hz. Nineteen electrodes were positioned according to the International 10–20 system. Most EEGs were recorded with alternating closed eyes, and the eyes were open for 3 min each, but some of the recordings only had closed eyes segments. The participants were alerted if they became visibly drowsy. The neurophysiology assistant recording the EEG made notations in the EEG when the participant closed and opened their eyes. After the recording, the files were exported as raw EEG.

### Collection and Analysis of Cerebrospinal Fluid

Lumbar puncture was performed between the L3/L4 or the L4/L5 intervertebral space, and the subsequent CSF was collected in polypropylene tubes. The analyses included routine parameters and the core AD biomarkers, i.e., Aβ_42_, T-tau, and P-tau. The AD biomarkers were quantified with sandwich ELISAs [INNOTEST amyloid-β_42_, hTau, and Phospho-Tau (181P), respectively; Fujirebio Europe, Ghent, Belgium]. The AD biomarker analyses from both clinics were all carried out at one central laboratory.

In some of the patients, the CSF concentration of the measured AD biomarker was above the detection rate. In these cases, the value was excluded from further analysis.

### Preprocessing of EEG

For the pre-processing steps, MATLAB (Mathworks, v2016a) and EEGLAB toolbox (Delorme and Makeig, 2004) were used. The closed eyes segments were selected and the electrodes were computationally located on the scalp using the dipfit toolbox (Oostenveld et al., 2011) using the standard 10–20 electrode model. Afterward, the data were bandpass-filtered from 1 to 70 Hz and bandstop-filtered from 45 to 55 Hz using the *pop_firws* function in MATLAB, with a filter order of 2, and the Kaiser window parameter beta was estimated using a maximum passband ripple of 0.001; the data were downsampled to 200 Hz. Afterward, the data were divided into 1-s epochs, and the epochs with excessive noise or artifacts as judged by visual inspection were removed. Spherical interpolation was applied for channels with excessive noise, drift, or bad connection. The EEG had to have less than or equal to three electrodes with excessive artifact; otherwise, the EEG was excluded from the analysis. Afterward, the EEGs were re-referenced to average reference, and independent component analysis was performed using the extended infomax algorithm (Lee et al., 1999) for each file. The components that contained eye blinks, eye movement, or specific line noise artifacts were removed. Lastly, the EEGs were inspected visually again, and the epochs with excessive noise or artifacts were removed. The investigator performing the preprocessing was blinded to the diagnosis.

### Cross-Frequency Coupling

To calculate CFC, we used the modulation index (MI) (Tort et al., 2008) since it has been shown to be less affected by noisy data recorded at a lower sampling compared with other techniques (Hülsemann et al., 2019). Since MI is affected by small segments, we concatenated the 70 1-s epochs of EEG from each participant, thereby having one epoch of 70 s. The number of epochs was selected based on the largest number of epochs being included while excluding the least number of participants. First, raw data are filtered at the two frequency ranges using the *pop_eegfiltnew* function from EEGLAB (Delorme and Makeig, 2004). Afterward, the Hilbert transform was applied to extract the time series of the amplitude of the higher frequency band and the phase of the lower frequency band. The composite time series is then constructed, which gives the amplitude of the higher frequency band oscillation at each phase of the lower frequency band rhythm. Next, the phases of the lower band were binned into 18 frequency bins, and the mean of the amplitude of the higher frequency band over each phase bin was calculated. We then normalize the mean amplitudes by dividing each bin value by the sum of the mean amplitudes over all the bins. Lastly, we calculated the associated entropy measure for the normalized mean amplitudes and used them to compute the MI (see the Supplementary Material for the MATLAB code). First, we calculated the gamma/theta CFC with gamma between 30 and 40 Hz and theta between 4 and 7 Hz. We did not include 40–50 Hz gamma due to contamination with line noise. For the exploratory analysis, we performed CFC for gamma–beta, gamma–alpha, gamma–delta, beta–alpha, beta–theta, beta–delta, alpha–theta, alpha–delta, and theta–delta. The MATLAB script used for calculating CFC in the current study can be found in the Supplementary Material.

### Statistics

All statistics were performed in MATLAB (vR2017b). When comparing the demographics and the cognitive scores for AD, MCI, and HC, we performed one-way ANOVAs, and *t*-tests were used to compare AD and HC, AD and MCI, and MCI and HC. The *t*-tests were used when comparing baseline demographics, cognitive test scores, and CSF measurements for pMCI and sMCI and for comparing baseline and 2nd-year follow-up for HC, MCI, and AD.

The CFC data were log-transformed before any of the subsequent analyses between groups due to the non-normal distribution. For comparing CFC between all three groups, we performed an ANCOVA (Gruner, 2020) with age, gender, education, and current medication as covariates. For comparing pMCI vs. sMCI based on the 2nd-year follow-up, we used a general linear model with the same covariate as mentioned above. In addition, we also compared AD and pMCI using the same technique as mentioned above. We performed correction for multiple comparisons for the electrode-to-electrode comparisons for each frequency band separately using false discovery rate. The comparisons between three groups and two groups for global CFC were computed as described above. For the correlation between gamma/theta CFC and CSF biomarkers (amyloid, total tau, and phosphorylated tau), ACE, and MMSE in patients with MCI, we used partial correlation with the same covariates as described above due to variability between subjects. The same analysis was performed to correlate ACE and total tau.

## Results

### Demographics

See Table 1 for characterization of the patients included in the analysis and the comparisons between groups. We found a significantly lower age for HC compared to MCI (*p*-value = 0.002, *t*-value = 3.187), and the mean MMSE was significantly lower in patients with AD (*p*-value < 0.001, *t*-value = −4.840) and MCI (*p*-value < 0.001, *t*-value = −4.646) compared to HC. Regarding medications, patients with MCI received significantly more antidepressants than HC (*p*-value < 0.05), while patients with AD received significantly more cholinesterase inhibitors (*p*-value < 0.001) compared to both HC and MCI. In AD, we found that CSF amyloid was significantly lower than in both MCI (*p*-value = 0.015) and HC (*p*-value < 0.001) and that it was significantly lower in MCI compared to HC (*p*-value 0.022). Furthermore, AD had a significantly higher concentration of CSF total tau compared to both MCI (*p*-value = 0.004) and HC (*p*-value < 0.001), and MCI showed a higher CSF total tau compared to HC (*p*-value = 0.012). The performance on cognitive screening instruments for baseline and at follow-up visit after 2 years with comparison between the scores can be seen in Table 2.

**TABLE 1:** Characteristics of the participants included in the analysis.

	HC (*n* = 36)	AD (*n* = 15)	MCI (*n* = 25)	*p*-value
Mean age (SD), years	65.8 (7.1)	70.1 (7.8)	71.4 (6.0)	0.008*
Female gender, *n*	16	8	6	0.133
Education, years (SD)	12.8 (3.6)	12.1 (4.0)	10.6 (3.4)	0.080
MMSE, mean (SD)	29.1 (1.0)	26.3 (3.2)	27.6 (1.5)	< 0.001*
Antidepressants	0	1	4	0.046*
Cholinesterase Inhibitors	0	8	1	< 0.001*
Pain killers	2	0	2	0.555
CSF amyloid, mean (SD)	997.5 (320.2)	550.7 (141.2)	782.3 (319.8)	< 0.001*
CSF total tau, mean (SD)	303.3 (144.7)	618.4 (186.0)	419.6 (173.9)	< 0.001*
CSF phosphorylated tau, mean (SD)	68.5 (103.4)	93.0 (33.3)	59.4 (21.5)	0.384

*HC, healthy controls; AD, Alzheimer’s disease; MCI, mild cognitive impairment; SD, standard deviation; MMSE, Mini-Mental State Examination; CSF, cerebrospinal fluid. The asterisk indicates a significant p-value (<0.05).*

**TABLE 2:** The cognitive scores, number of participants that dropped out, and number of patients with mild cognitive impairment (MCI) that progressed during follow-up in year 2.

	Baseline	2nd-year follow-up	*t*-value	*p*-value
**HC**				
Dropout/total (*n*)	0	1/36		
Progression/no-progression	NR	NR		
MMSE, mean (SD)	29.37 (0.84)	29.08 (1.00)	–1.313	0.193
ACE, mean (SD)	95.66 (3.34)	94.72 (3.33)	–1.181	0.242
MDI, mean (SD)	3.86 (3.13)	3.53 (2.85)	–0.464	0.644
**MCI**				
Dropout/total (*n*)	0	6/25		
Progression/no-progression	NR	12/13		
MMSE, mean (SD)	27.60 (1.50)	26.00 (3.33)	2.138	0.038*
ACE, mean (SD)	84.13 (8.17)	79.67 (11.59)	1.464	0.151
MDI, mean (SD)	7.13 (5.91)	10.22 (7.75)	–1.450	0.155
NPI, mean (SD)	3.38 (3.49)	5.24 (2.49)	–1.844	0.073
ADL, mean (SD)	70.71 (4.84)	66.59 (9.87)	1.544	0.133
**AD**				
Dropout/total (*n*)	0	7/15		
Progression/no-progression	NR	NR		
MMSE, mean (SD)	26.27 (3.17)	23.50 (5.53)	1.537	0.139
ACE, mean (SD)	77.60 (12.87)	67.14 (18.85)	1.532	0.141
MDI, mean (SD)	5.67 (4.70)	4.17 (4.62)	0.664	0.515
NPI, mean (SD)	1.5 (1.24)	5.00 (2.45)	–4.235	< 0.000*
ADL, mean (SD)	70.86 (8.16)	67.38 (8.67)	0.942	0.358
Missing values (%)	6.78	26.21		

*In addition, the percentage of missing values for the cognitive scores can be seen. All cognitive scores have been compared over time using a paired *t*-test. The asterisk indicates a significant *p*-value (<0.05).*

When comparing the scores at baseline between pMCI and sMCI, we found that ACE was significantly different (see Table 3). When looking at the ACE subscores, we found that the ACE subscore comprehension was significantly different between the two groups [mean pMCI (SD) = 3.57 (0.79), mean sMCI (SD) = 4 (0), *p*-value = 0.035, *t*-stat = −2.249] and a trend for anterograde memory [mean pMCI (SD) = 16.14 (7.71), mean sMCI (SD) = 21.50 (4.68), *p*-value = 0.051, *t*-stat = −2.070]. The rest of the subscores were not significantly different between pMCI and sMCI (*p*-value > 0.05).

**TABLE 3:** Demographics, baseline cognitive scores, and cerebrospinal fluid results for stable mild cognitive impairment (sMCI) and progressed mild cognitive impairment (pMCI).

	Baseline – sMCI (*n* = 13)	Baseline – pMCI (*n* = 11)	*p*-value
Mean age (SD), years	72.38 (6.06)	70.27 (6.63)	0.424
Female gender, *n*	4	2	0.500
Education, years (SD)	10.69 (3.84)	10.55 (3.36)	0.922
CSF amyloid, mean (SD)	820.08 (348.64)	695.75 (309.90)	0.419
CSF total tau, mean (SD)	398.25 (162.10)	461.56 (206.29)	0.440
CSF phosphorylated tau, mean (SD)	60.54 (24.54)	59.89 (19.28)	0.948
MMSE, mean (SD)	27.92 (1.38)	27.09 (1.58)	0.182
ACE, mean (SD)	87.54 (6.08)	79.00 (8.36)	0.010*
MDI, mean (SD)	8.67 (6.89)	6.00 (4.14)	0.297
NPI, mean (SD)	3.09 (3.96)	3.00 (2.24)	0.952
CDR, mean (SD)	0.50 (0)	0.56 (0.17)	0.281
ADL, mean (SD)	70.60 (6.06)	70.86 (2.73)	0.918

*The *t*-tests were performed to compare the two groups for each score separately. The asterisk indicates a significant (*p*-value < 0.05) difference. One patient with mild cognitive impairment showed up during follow-up to fulfill the criteria for vascular dementia and was not included in the comparison between pMCI and sMCI.*

### Gamma/Theta Cross-Frequency Coupling

**FIGURE 1:**
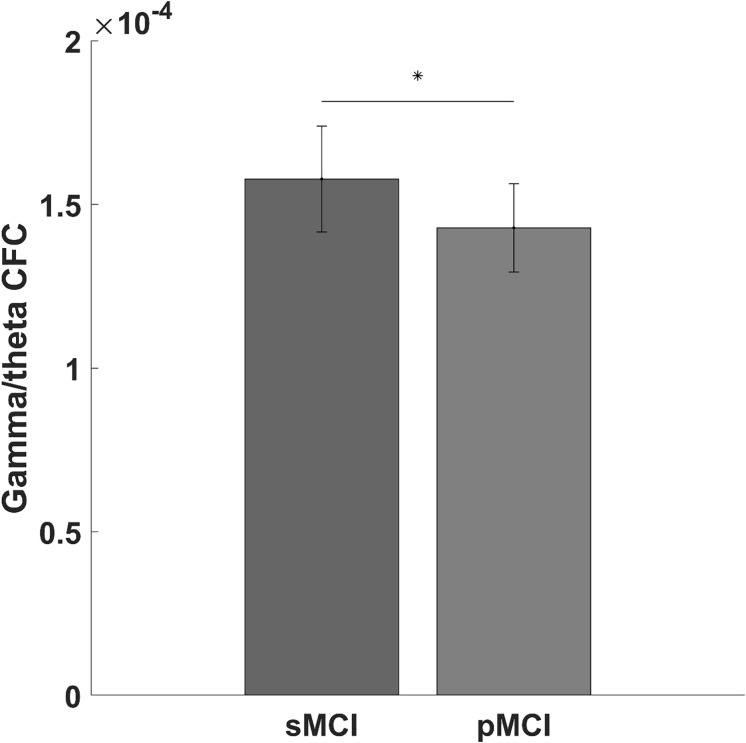
Bar graph showing the gamma/theta cross-frequency coupling for progressed mild cognitive impairment (pMCI) or stable mild cognitive impairment (sMCI). The star indicates a significant difference between pMCI and sMCI (*p*-value = 0.004, *t*-value = –11.09).

**FIGURE 2:**
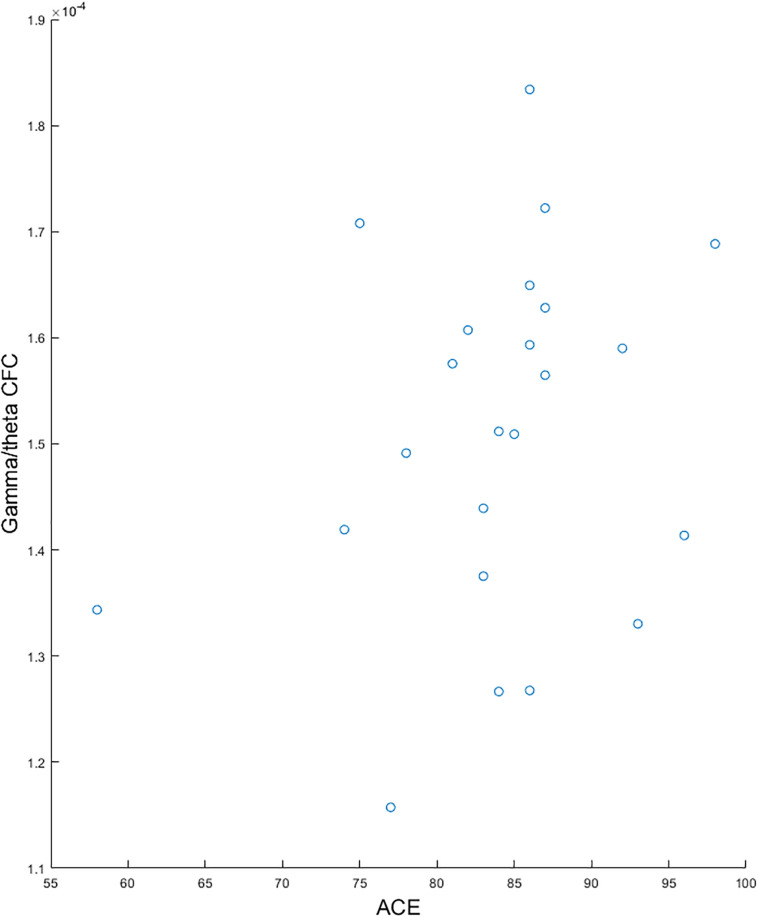
Scatterplots showing the significant correlation between gamma/theta cross-frequency coupling and Addenbrooke’s Cognitive Examination for patients with mild cognitive impairment.

We found a significantly lower global gamma/theta CFC in pMCI compared with sMCI (*p*-value = 0.004, *t*-value = −11.09; see Figure 1). After adjusting for multiple comparisons using false discovery rate, no significant differences in the electrode-to-electrode comparisons between pMCI and sMCI were found. The strongest correlation was found between ACE and gamma/theta CFC (*p*-value = 0.030, rho = 0.527; see Figure 2 for the scatterplot). No significant correlations were found between gamma/theta CFC and amyloid (*p*-value = 0.971, rho = 0.010), total tau (*p*-value = 0.592, rho = −0.151), or phosphorylated tau (*p*-value = 0.824, rho = −0.060) in MCI. Since total tau is a marker for neurodegeneration and found to be related to progression from MCI to AD (Diniz et al., 2008), we investigated the partial correlation between ACE and total tau in patients with MCI, which was not significant (*p*-value = 0.107, rho = −0.449).

**FIGURE 3:**
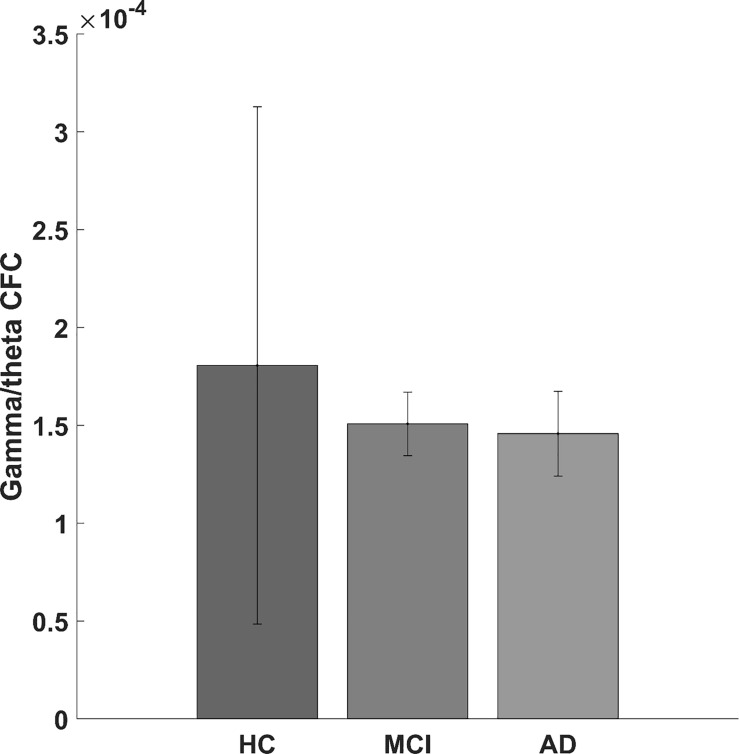
Bar graph showing the gamma/theta cross-frequency coupling for healthy controls and for mild cognitive impairment and Alzheimer’s disease patients.

When looking at AD and MCI compared to HC, we found a significantly lower gamma/theta CFC for patients with AD and MCI compared with HC (adjusted *p*-value < 0.05). Although not significant (*p*-value = 0.170, *F*-value = 1.821), we found a lower gamma/theta CFC in AD and MCI compared with that in HC (see Figure 3).

When comparing the global gamma/theta CFC between AD and pMCI, we did not find a significant difference (*p*-value = 0.555, *F*-value = 0.360).

### Exploratory Cross-Frequency Coupling Analyses

**FIGURE 4:**
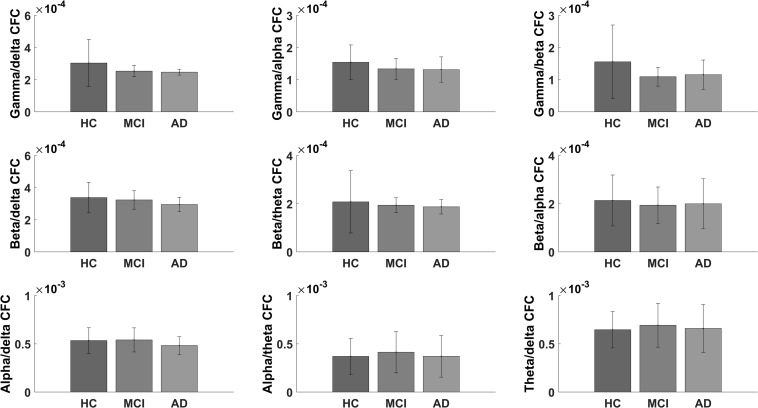
Bar graph showing the global scores for healthy controls and for mild cognitive impairment and Alzheimer’s disease patients for the exploratory cross-frequency coupling analyses.

When examining the difference in CFC between multiple frequency bands, we did not find any significant changes in the electrode-to-electrode comparisons after correcting for multiple comparisons using false discovery rate (*p*-value < 0.05). The largest difference in global CFC was found for gamma/alpha CFC (*p*-value = 0.076, *F*-value = 2.678; see Figure 4 for the bar plots of global CFC). When comparing the global CFC for the rest, the differences were not as pronounced (*p*-value > 0.1).

## Discussion

In the current study, we found that global gamma/theta CFC was significantly lower in pMCI compared to that in sMCI. Furthermore, gamma/theta CFC was strongly correlated with ACE (*p*-value = 0.030, rho = 0.527). In the exploratory analysis of CFC, global gamma/alpha CFC showed the largest difference between the groups.

Only a few studies have investigated the changes in CFC in patients with AD. Here, two of the studies have found decreased CFC (Poza et al., 2017; Goodman et al., 2018), while one study found increased CFC (Wang et al., 2017) in patients with AD compared to HC using the amplitude at a high-frequency band and the phase at a low-frequency band. In the current study, we found, although not significant, that gamma/theta CFC was decreased in both patients with AD and MCI compared to that in HC. This finding suggests that patients with AD show a disruption of the coupling between the frequency bands. More interestingly, we found that gamma/theta CFC was decreased in pMCI compared to sMCI. Gamma/theta CFC has been associated with activity in the hippocampus and performance in memory tasks in rodents (Tort et al., 2009; Canolty and Knight, 2010; Lisman and Jensen, 2013) and has been shown to be vital for working memory in humans (Axmacher et al., 2010). This could suggest a disruption of brain areas known to be affected in AD. We hypothesize that the decreased gamma/theta CFC in pMCI is associated with a network dysfunction but possibly not directly associated with atrophy since no correlation was found with total tau. The lack of any electrode-specific changes between pMCI and sMCI could indicate that this is more related to global dysfunction and less specifically to the temporal lobes. In addition, studies implementing either an N-back task (Goodman et al., 2018) or an auditory oddball paradigm (Dimitriadis et al., 2015) found more pronounced results, which may indicate that the difference in CFC is larger during a task. As a diagnostic tool in AD and MCI, this suggests that gamma/theta CFC should be implemented during a task. Other EEG markers of progression have been suggested, and decreased spectral beta power has been the most commonly reported (Jelic et al., 2000; Poil et al., 2013; Musaeus et al., 2018b). This study suggests that gamma/theta CFC may serve as a marker of progression but has some limitations for clinical application due to low signal-to-noise ratio in the gamma band and the possible necessity to conduct the EEG during a task. More studies are needed to understand the applicability of gamma/theta CFC, which may, however, be beneficial in research on the pathophysiology of progressive neurodegenerative disorders vs. non-progressive conditions and potentially also in clinical trials.

When investigating the relationship between global gamma/theta CFC and clinical presentation, we found that it was correlated with ACE (*p*-value = 0.030, rho = 0.527). Previous studies have found that gamma/theta CFC has been associated with dysfunction in the temporal lobe structures. However, since we calculated the global gamma/theta CFC score, it may be the reason for a significant correlation with ACE since it is a score for multiple cognitive domains (Mathuranath et al., 2000). Therefore, gamma/theta CFC may reflect decreased functional coupling between the temporal lobes and the rest of the brain. Total tau has previously been shown to be a marker progression from MCI to AD (Diniz et al., 2008) and associated with neurodegeneration regardless of the underlying condition (Jack and Holtzman, 2013) and may therefore be related to gamma/theta CFC. However, we did not find a significant correlation between total tau and gamma/theta CFC nor total ACE test score. This may be attributed to the low sample size or delays between neurodegeneration and subsequent changes in cognition. If gamma/theta CFC is to be considered as an early marker of progressive symptoms, it may also be abnormal in patients with progressive pathophysiology with yet no neurodegeneration, parallel to what is observed in, for instance, PET biomarker studies.

The majority of studies have focused on gamma/theta CFC, but coupling between other frequency bands has been shown to play an important role for coordination in perception, consciousness, and working memory (Palva and Palva, 2007) and has been associated with neuropsychiatric symptoms (Miskovic et al., 2010), which are also prevalent in patients with AD. However, we did not find any significant changes for CFC between AD, MCI, and HC in the other frequency bands. The largest difference was found for gamma/alpha coupling (*p*-value = 0.076, *F*-value = 2.678). The lack of findings suggests that gamma/theta coupling may be the most important measure in AD. However, larger studies are needed to fully understand the pathophysiological role of CFC in AD.

When examining the neuropsychological test scores between pMCI and sMCI, we found that patients with pMCI had a significantly lower ACE compared to patients with sMCI (see Table 3). Studies have previously found that neuropsychological tests (Maioli et al., 2007; Rozzini et al., 2008) and ACE (Galton et al., 2005; Mitchell et al., 2009) are able to predict the progression from MCI to AD. Furthermore, we found that global gamma/theta CFC was not significantly different between pMCI and AD. These findings may indicate that the included patients with pMCI were further downstream in the disease compared to patients with sMCI at the time of inclusion or that pMCI and sMCI comprised a fundamentally different underlying etiology. More studies are needed to investigate the potential of CFC as a marker of progression and potentially include patients at a very early stage of the disease.

The current study has some limitations. Firstly, we acknowledge the relatively small sample size, but these changes suggest an overall decoupling between the frequency bands even in the early stages of AD. Furthermore, we found a significant difference in the age between HC and MCI and have therefore used age as a covariate in the analysis. In addition, the follow-up time was short and, according to previous studies, the annual clinical progression rate is 15% (Petersen et al., 1999; Saxton et al., 2009), which means that only 30% of the patients with MCI should have progressed to AD in the present cohort. However, we found that 48% progressed, which may in part be due to the patients with MCI being at a more advanced stage of the disease at inclusion. Furthermore, we concatenated 1-s epochs and, due to the discontinuity, this may have induced some additional noise to the calculations. Due to line noise, we calculated the amplitude of gamma between 30 and 40 Hz, which is a narrow range compared to the previous studies analyzing CFC (Canolty and Knight, 2010). In addition, the gamma band for standard scalp EEG has been suggested to be highly influenced by muscle activity (Whitham et al., 2007, 2008), and this poses some limitations in implementing this technique as a clinical tool. Nevertheless, our findings in this small pilot study with decreased global gamma/theta CFC in patients with pMCI compared to patients with sMCI may be able to guide larger studies.

## Conclusion

In conclusion, our findings suggest that decreased global gamma/theta CFC is associated with patients with MCI, who over time progress to AD. Furthermore, global gamma/theta CFC may be related to global cognitive function as assessed with ACE. Gamma/theta CFC may therefore serve as a progression marker in MCI. However, larger studies are needed to validate these findings.

## Data Availability Statement

The datasets supporting the conclusions of this article will be made available by the authors to any qualified researcher. However, due to regulations, we are not able to share the EEG files. Requests to access the datasets should be directed to CM, christian.sandoee.musaeus@regionh.dk.

## Ethics Statement

The studies involving human participants were reviewed and approved by the Regional Committee on Health Research Ethics. The patients/participants provided their written informed consent to participate in this study.

## Author Contributions

PH, MN, and CM conceived the project idea of using quantitative EEG. PH and MN conducted the experiments. CM and JM conducted the data analyses. CM drafted the manuscript. PH, MN, JM, and CM contributed to revising the manuscript. All authors contributed to the article and approved the submitted version.

## Conflict of Interest

The authors declare that the research was conducted in the absence of any commercial or financial relationships that could be construed as a potential conflict of interest.
