# Supplementary material for: Electroencephalographic Cross-Frequency Coupling as a Sign of Disease Progression in Patients With Mild Cognitive Impairment: A Pilot Study
Source: Front Neurosci. 2020 Aug 11;14:790. doi: 10.3389/fnins.2020.00790 (PMC7431634; doi:10.3389/fnins.2020.00790)
Supplement: Supplementary file 1 [file Table_1.DOCX]

**Supplementary material**

The code for calculating CFC based on the modulation index (Tort et al. 2008). The inputs for this function is the filtered EEG for the amplitude frequency band (x_fA) and for the phase frequency band (x_fp) and the number of angular bins (N).

%% Function to compute modulation index as described in Tort et al. 2008

% Written by Jørgen Sandøe Musaeus and Christian Sandøe Musaeus

function [MI] = ComputeMI(x_fA,x_fp,N)

% Sample count and number of electrodes

[C,S] = size(x_fA);

% Hilbert transformation

Hx_fp = hilbert(x_fp);

Hx_fA = hilbert(x_fA);

% Finding angle and amplitude to the corresponding frequency bands

phi = angle(Hx_fp);

A = abs(Hx_fA);

% Size of the angular bins

dp = 2*pi/N;

% Finding the average amplitude within each angular bin

A_ave = zeros(C,N,2);

for i = 1:S

for j = 1:C

if phi(j,i,1) >= 0

k = ceil(phi(j,i,1)/dp);

else

k = ceil((2*pi+phi(j,i,1))/dp);

end

A_ave(j,k,1) = A_ave(j,k,1)*A_ave(j,k,2)/(A_ave(j,k,2)+1) + A(j,i,1)/(A_ave(j,k,2)+1);

A_ave(j,k,2) = A_ave(j,k,2) + 1;

end

end

% Computing probabilities for each angular bin

prob = zeros(C,N);

for i = 1:C

for j = 1:N

prob(i,j) = A_ave(i,j,1)/sum(A_ave(i,:,1));

end

end

% Computing the entropy

H = zeros(C,1);

for i = 1:C

for j = 1:N

if prob(i,j) > 0

H(i) = H(i) - prob(i,j)*log(prob(i,j));

end

end

end

% Computing the modulation index

MI = zeros(C,1);

for i = 1:C

MI(i) = 1 - H(i)/log(N);

end

end
